# Lingual Leishmaniasis Presenting to Maxillofacial Surgery in UK with Successful Treatment with Miltefosine

**DOI:** 10.1155/2013/975131

**Published:** 2013-09-30

**Authors:** K. Kassam, R. Davidson, P. J. Tadrous, M. Kumar

**Affiliations:** Department of Oral & Maxillofacial Surgery, Head and Neck Surgery, Northwick Park Hospital, HA1 3UJ London, UK

## Abstract

Leishmaniasis is a disease that is caused by protozoa of the genus *Leishmania*, which is prevalent in tropical and subtropical areas. Clinical forms of leishmaniasis are particularly diverse representing a complex of diseases. We present a case of lingual Leishmaniasis in an immunocompetent man. The lesions were caused by *Leishmania donovani/infantum* species. The patient responded excellently to miltefosine treatment, with no reactivation during followup. To the authors' knowledge, it is the first such case of successful miltefosine treatment in this unusual variant of leishmaniasis occurring on the tongue.

## 1. Case Report

**Figure 1:**
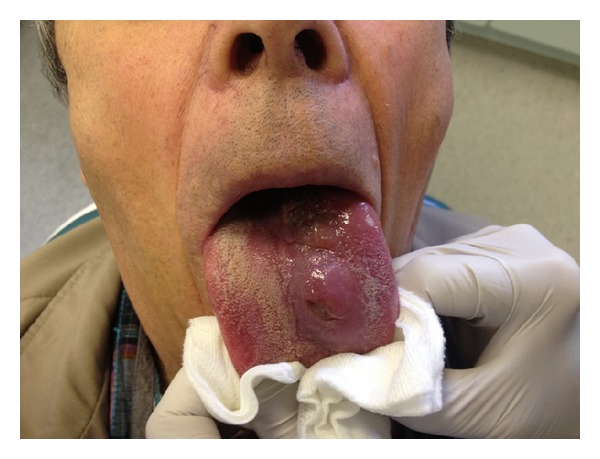
Presentation of tongue lesion.

A 66-year-old patient was referred by his GP with a 3-month history of swelling and soreness of the dorsal aspect of the tongue (Figure 1). He had been referred in as an urgent 2-week wait patient as he had ulceration for more than 3 weeks. His complaint was of painful ulceration and some difficulty in swallowing. His past medical history included chronic obstructive airways disease diagnosed in 2002 and had recently had 3 courses of antibiotics for acute exacerbations for the condition. He was an ex-smoker with a 40-pack year history and did not drink alcohol. It was noted in the last 3 months his breathlessness had deteriorated markedly with no obvious precipitating event. His exercise tolerance was severely limited at 10 steps. His usual medication included salbutamol and steroid inhalers and an antihistamine. There was no history of fevers, recent travel (especially to South America), or another swellings or lymph nodes in the body. 

On examination, there was no extraoral lymphadenopathy, but intraoral examination revealed firm swelling on the left mid dorsum of tongue with lymphoid-like tissue swelling. Differential diagnosis at this point included squamous cell carcinoma, tuberculosis, and lymphoma. 

An urgent biopsy was arranged along with relevant blood tests. 

## 2. Results

Blood tests were unremarkable apart from a raised ESR (34 mm/hr).

Hepatitis B and C and HIV tests were also carried out, which were negative.

## 3. Histopathology

**Figure 2:**
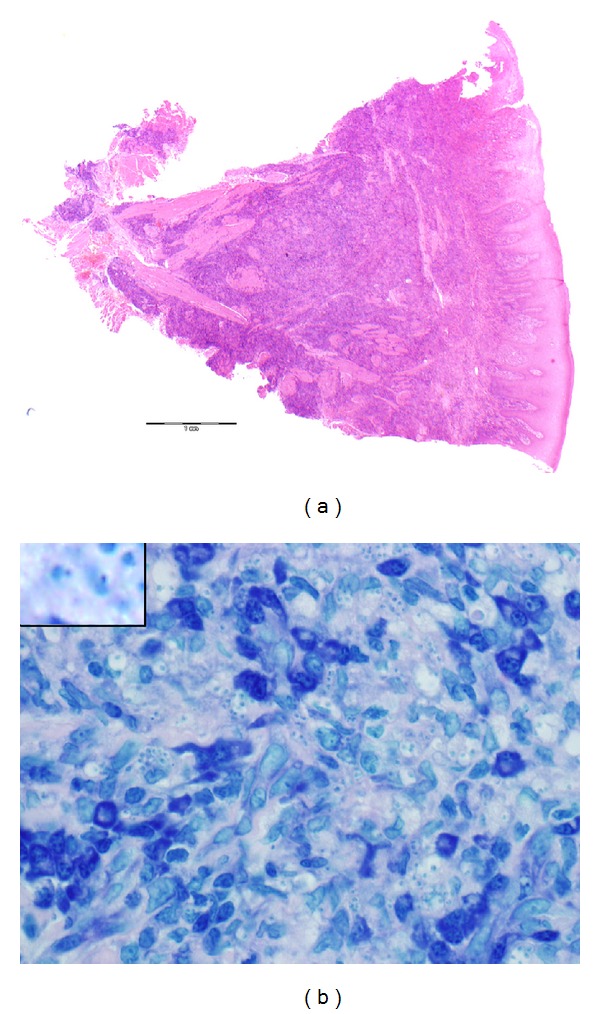
Histopathology. (a) shows a low-power (×20) view of a cross section of the initial tongue biopsy stained with H and E and demonstrates the intense histiocyte-rich inflammatory infiltrate, sparing the squamous epithelium (right) and almost filling the corium extending deep into the soft tissues. (b) shows an oil immersion high-power view of the histiocytic infiltrate (×1000) stained with Giemsa and shows the histiocytes with many intracellular amastigote forms characterized by the “double-dot” sign (inset ×4 compared to the original). These are the Leishman-Donovan bodies.

Histology showed a histiocyte-rich infiltrate. The histiocytes contained numerous inclusions of Leishman-Donovan bodies indicative of Leishmaniasis. They have a double-dot pattern with Giemsa as shown in the figure; the larger ovoid dot is the nucleus and the smaller dot is the kinetoplast of the amastigote organism (Figure 2).

The diagnosis is clear from the morphology but was also later confirmed by positive PCR on a subsequent biopsy showing *Leishmania donovani* complex DNA. 

One week after biopsy, the patient complained of increased difficulty in swallowing and voice change. Urgent flexible nasal endoscopy was carried out in conjunction with MRI scanning of the head and neck. Apart from mild supraglottic oedema, the examinations were normal.

### 3.1. Treatment

**Figure 3:**
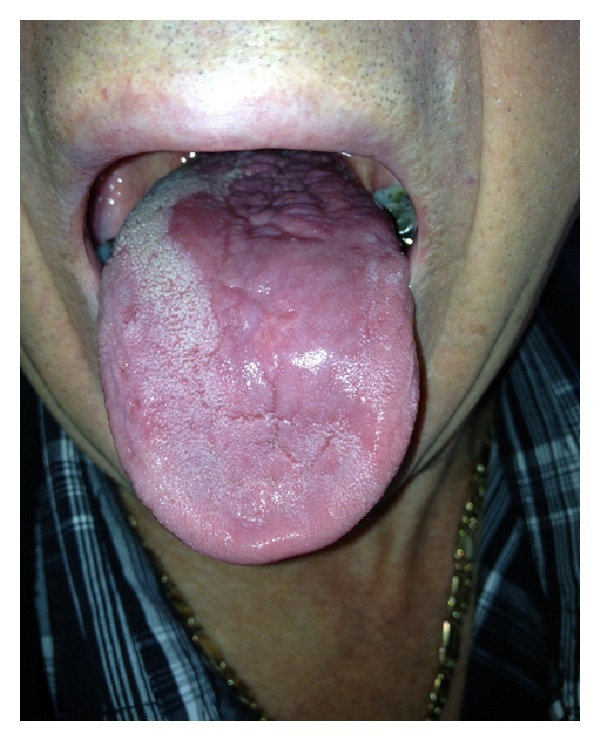
Three months after treatment.

The patient was commenced on 150 mg BD miltefosine for 28 days and responded very well to the treatment. The mouth was no longer sore and the lesions resolved (Figure 3). In addition, his acute respiratory problems also seemed to resolve.

## 4. Discussion

Miltefosine, an alkylphospholipid, was developed as an oral antineoplastic agent (for cutaneous cancers) and has subsequently been applied to treat leishmaniasis. It interferes with cell signal transduction pathways and inhibits phospholipids and sterol biosyntheses.

The discovery that miltefosine is effective against *Leishmania* led to the identification of a modern group of antiprotozoal medicines [1]. Following clinical studies, miltefosine was approved as Impavido and has become the first oral treatment for leishmaniasis in some countries [2]. It is an effective treatment for visceral and cutaneous leishmaniasis, including antimony-resistant infections [3]. Miltefosine was registered in India for the treatment of visceral leishmaniases in 2002 [4]. 

Lingual leishmaniasis is very uncommon, and its optimal treatment is not known. It has been reported in transplant patients [5, 6] and in HIV patients [7]. In our case, our patient did use intermittent inhaled corticosteroids which may have contributed to his eventual pathology. 

The European parasite, *L. infantum*, more commonly produces visceral leishmaniasis with parasites in liver, spleen, and marrow (but without oral lesions), and in such cases liposomal amphotericin B is the drug of choice preference; its high concentrations in the macrophage-rich tissues enable low total doses, which offset its high cost. Our case resembled mucocutaneous leishmaniasis more closely than visceral leishmaniasis. We chose to use miltefosine rather than IV sodium stibogluconate or liposomal amphotericin B, for several reasons. First was the fact that miltefosine has proven efficacy in mucocutaneous leishmaniasis, and its oral route enables outpatient care, which is economic for the hospital and convenient for the patient. Second, sodium stibogluconate, which has extensive historical use in *L. infantum* visceral leishmaniasis, including some cases of lingual disease, has common adverse effects, of which pancreatitis is the most common and cardiac arrest is the most serious; it requires inpatient care. Third, total doses of liposomal amphotericin B of 40–60 mg per kg are recommended for patients with mucocutaneous leishmaniasis [8], which is extremely expensive. Our patient had a favourable response to miltefosine, with no adverse effects.

To the authors' knowledge, this is the first case of lingual leishmaniasis treated by miltefosine. There has been no recurrence to date, at 10 months. Miltefosine has the advantage of use in the outpatient setting as it can be administered orally unlike most of the antileishmaniasis drugs. Leishmaniasis is rarely seen in UK; however, it should be in the differential diagnosis of the clinician with increased travel.
